# Twin Presentation of Cutaneous Neonatal Lupus Associated With Maternal Anti-U1RNP Antibodies: A Case Report

**DOI:** 10.7759/cureus.101451

**Published:** 2026-01-13

**Authors:** Sofia Guedes, Patrícia Veríssimo, Ana T Maria, Mónica Marçal

**Affiliations:** 1 Pediatrics Department, Neonatal Intensive Care Unit, Hospital de São Francisco Xavier, Unidade Local de Saúde Lisboa Ocidental, Lisbon, PRT; 2 Pediatrics Department, Hospital de Santarém, Unidade Local de Saúde da Lezíria, Santarém, PRT; 3 Neonatology Unit, Child and Adolescent Center, CUF Descobertas Hospital, Lisbon, PRT

**Keywords:** anti-rnp antibodies, catheter, neonatal lupus, skin, twins

## Abstract

Neonatal lupus erythematosus (NLE) is a rare autoimmune condition caused by the transplacental transfer of maternal IgG autoantibodies, most commonly anti-Sjögren's-syndrome-related antigen A (anti-Ro/SSA) and anti-Sjögren’s-syndrome-related antigen B (anti-La/SSB). NLE associated solely with anti-U1RNP antibodies is uncommon and typically limited to cutaneous manifestations, with extremely few cases reported in twins. We describe bi-chorionic bi-amniotic preterm twin girls who developed atypical erythematous lesions along the trajectory of epicutaneo-cava catheters during the second week of life. Autoimmune screening revealed strongly positive anti-U1RNP antibodies in both infants; cardiac evaluation was normal. Autoantibodies became negative by eight months. Both twins showed normal development and no further manifestations at 15 months. Maternal evaluation uncovered previously unrecognized antinuclear antibody (ANA) and anti-ribonucleoprotein antibody(anti-RNP) positivity. This appears to be the first reported case of anti-U1RNP NLE presenting with catheter-aligned inflammatory lesions in twins. The case highlights the role of trauma-induced Koebnerization as a potential trigger and underscores the importance of considering autoimmune etiologies in synchronous neonatal skin lesions, as well as the value of neonatal findings in identifying occult maternal autoimmunity.

## Introduction

Neonatal lupus erythematosus (NLE) is a rare, passively acquired autoimmune disorder resulting from the transplacental transfer of maternal immunoglobulin G (IgG) autoantibodies [1,2]. Its estimated prevalence is approximately one in 12,500-20,000 live births [3]. Anti-Sjögren’s-syndrome-related antigen A (anti-Ro/SSA) and anti-Sjögren’s-syndrome-related antigen B (anti-La/SSB) antibodies are the main immunologic markers implicated in its pathogenesis [2-4]. NLE is characterized primarily by cardiac and cutaneous manifestations, although hematologic abnormalities (anemia, neutropenia, thrombocytopenia) and hepatobiliary involvement may also occur [1,3,5]. The most serious complication is third-degree atrioventricular (AV) block, which is typically permanent. In contrast, non-cardiac findings are transient and resolve as maternal antibodies are cleared from the infant’s circulation, generally by six to eight months of age [1-3,6]. Importantly, up to 60% of mothers of affected infants are asymptomatic at diagnosis, and as many as half may subsequently develop features of an autoimmune disease [2].

Although anti-Ro/SSA and anti-La/SSB antibodies remain the most frequently associated autoantibodies, neonatal lupus may also occur in the presence of anti-ribonucleoprotein (anti-RNP) antibodies, particularly anti-U1RNP [2,3,7,8]. In these cases, the disease tends to be limited to the skin, and the cutaneous findings may be atypical compared with classic NLE presentations [7-10].

The diagnosis of neonatal lupus is established when two criteria are met: the detection of specific maternal or neonatal autoantibodies and the presence of characteristic clinical manifestations without another explanation [1,4,11]. The prognosis for NLE presenting solely with cutaneous manifestations associated with anti-RNP antibodies (in the absence of anti-Ro/SSA and anti-La/SSB antibodies) is generally considered excellent and benign. This specific subset of NLE is distinct from the more common forms primarily due to the typical absence of life-threatening cardiac involvement [7,8].

NLE is uncommon in twins, and published reports are scarce. To date, fewer than five cases involving twins have been described in the literature, and only one with isolated anti-U1RNP antibodies, presenting with typical cutaneous-only disease [12]. 

Here, we describe a distinctive case of cutaneous NLE occurring in bi-chorionic, bi-amniotic (BCBA) twins with positive anti-U1RNP antibodies. Uniquely, both newborns presented with erythematous lesions along the trajectory of an epicutaneo-cava catheter (ECC), which triggered the investigation. 

## Case presentation

Two preterm twin newborn girls were delivered at 33 weeks of gestation from a BCBA pregnancy complicated by gestational diabetes diagnosed in the first trimester, and medically managed with metformin and insulin. Serological tests during pregnancy were unremarkable. Third-trimester screening showed: Group B *Streptococcus* negative, rubella immune, hepatitis B surface antigen (HBsAg) negative, HIV 1/2 antibodies negative, HCV antibodies negative, and negative treponemal and toxoplasma serology. Prenatal ultrasound revealed fetal growth restriction beginning at 31 weeks (Fetus A: 0.6th centile; Fetus B: 1.2th centile). Delivery occurred by caesarean section due to pre-eclampsia. There was an intrapartum rupture of membranes with clear amniotic fluid.

Twin A had a breech presentation with birthweight 1386 g (3rd-10th centile), length 41 cm (10th-50th centile), and head circumference 27 cm (10th centile). Apgar scores were 8/9/9. She presented with grunting and received positive end-expiratory pressure (PEEP) via face mask, with a good response. Twin B also had a breech presentation, with birthweight 1336 g (3rd-10th centile), length 41 cm (10th-50th centile), and head circumference 28 cm (10th centile). Apgar scores were 8/9/9. She presented with respiratory distress syndrome and grunting, requiring PEEP via face mask, and also had a good response. 

**Figure 1:**
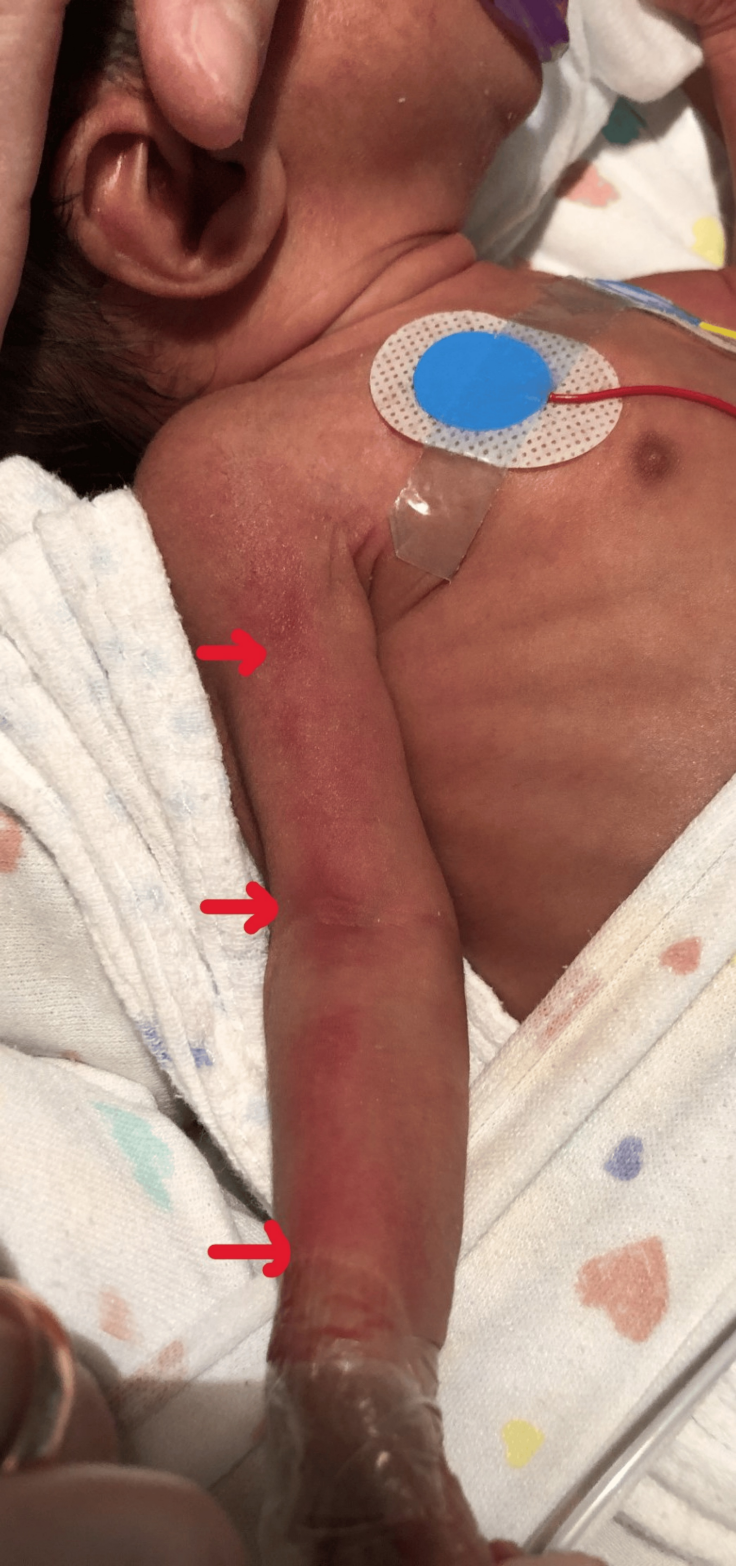
Erythematous rash along the epicutaneo-cava catheter in the right upper limb (Twin A)

**Figure 2:**
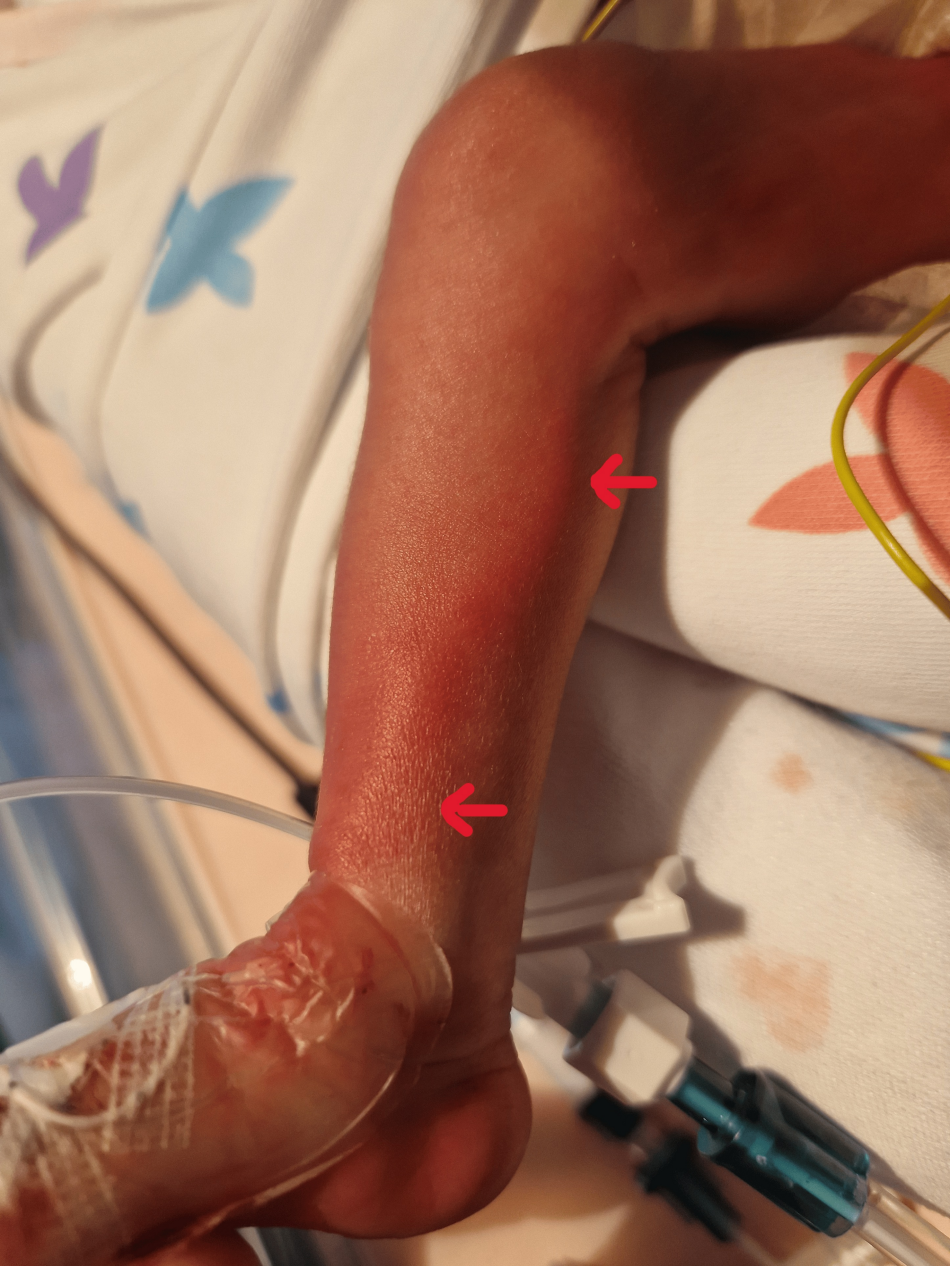
Erythematous rash along the epicutaneo-cava cathether in the right lower limb (Twin A)

Both twins were admitted to the neonatal intensive care unit (NICU). During admission, on day of life (DOL) 8, twin A developed an erythematous skin rash with inflammatory features along the trajectory of the ECC placed in the right upper limb (Figure 1). Given the suspicion of phlebitis, anti-staphylococcal antibiotic therapy was initiated, and the catheter was removed and replaced with a new one in the right lower limb. On DOL 12, she again developed inflammatory signs along the new catheter pathway (Figure 2). Microbiological evaluation included negative peripheral blood cultures on D9, D13, and D23, and antibiotics were discontinued after three days.

In parallel, on DOL 11, twin B developed inflammatory signs at the catheter insertion site and along the catheter trajectory on the right upper limb, which was interpreted as phlebitis. The catheter was removed the following day. She was started on flucloxacillin. Microbiological cultures revealed, in a catheter-drawn sample (D12), methicillin-susceptible *Staphylococcus aureus* and oxacillin/gentamicin-resistant *Staphylococcus haemolyticus*. Peripheral blood cultures (D12, D13, D15) were negative. She completed flucloxacillin for 14 days and gentamicin for 5 days. The inflammatory lesions resolved after catheter removal.

Because both infants presented similar cutaneous inflammatory changes, in the same timeline, and both were associated with the site of the ECC, an autoimmune investigation was performed. Both twins had strongly positive anti-U1RNP antibodies and antinuclear antibodies (ANA), with negative anti-double stranded DNA (dsDNA) antibodies and extractable nuclear antigen antibodies (ENA). Complement levels (C3, C4) and immunoglobulins (IgG, IgM, IgA) were within normal range (Table 1 and Table 2, respectively, for twins A and B). Notably, twin A had moderate neutropenia, which resolved within one week. Table 3 and Table 4 summarize the laboratory investigations performed in twins A and B, respectively, during admission and follow-up. Following these findings, both underwent cardiology screening, and their electrocardiograms were normal, excluding AV block. 

**Table 1:** Laboratory findings of Twin A at day of life 14, after the onset of the cutaneous rash. IgA: immunoglobulin A; IgG: immunoglobulin G; IgM: immunoglobulin M; DOL: day of life

Analytical Parameters	Patient Values (DOL 14)	Reference Ranges
Complement C3 (mg/dL)	100	90–180
Complement C4 (mg/dL)	15.5	10–40
IgG (mg/dL)	693	141–930
IgA (mg/dL)	0.66	5–64
IgM (mg/dL)	22.8	14–142

**Table 2:** Laboratory findings of Twin B at day of life 14, after the onset of the cutaneous rash. IgA: immunoglobulin A; IgG: immunoglobulin G; IgM: immunoglobulin M; DOL: day of life

Analytical Parameters	Patient Values (DOL 14)	Reference Ranges
Complement C3 (mg/dL)	101	90–180
Complement C4 (mg/dL)	23.7	10–40
IgG (mg/dL)	721	141–930
IgA (mg/dL)	2.39	5–64
IgM (mg/dL)	21.5	14–142

**Table 3:** Laboratory findings of Twin A during admission and follow-up. ALT: alanine aminotransferase; AST: aspartate aminotransferase; CRP: C-reactive protein; GGT: gamma-glutamyl transferase; DOL: day of life

Analytical Parameters	Patient Values (DOL 1)	Patient Values (8 months old)	Reference Ranges
Hemoglobin (g/dL)	18.7	12.0	10.5–18.5
Leukocytes (x10^9^/L)	4.7	9.1	8.0–26.0
Neutrophils (x10^9^/L)	0.80	1.14	1.0–14.0
Platelets(x10^9^/L)	199	344	150–550
CRP (mg/dL)	<0.10	<0.10	<0.50
AST (U/L)	–	36	<84
ALT (U/L)	–	18	<60
GGT (U/L)	–	11	6–42

**Table 4:** Laboratory findings of Twin B during admission and follow-up. ALT: alanine aminotransferase; AST: aspartate aminotransferase; CRP: C-reactive protein; GGT: gamma-glutamyl transferase; DOL: day of life

Analytical Parameters	Patient Values (DOL 1)	Patient Values (8 months old)	Reference Ranges
Hemoglobin (g/dL)	16.8	12.0	10.5–18.5
Leukocytes (x10^9^/L)	6.4	9.1	8.0–26.0
Neutrophils (x10^9^/L)	2.18	1.14	1.0–14.0
Platelets(x10^9^/L)	193	344	150–550
CRP (mg/dL)	<0.10	<0.10	<0.50
AST (U/L)	-	31	<84
ALT (U/L)	-	17	<60
GGT (U/L)	-	10	6–42

During the remainder of the hospital stay, from a respiratory perspective, both newborns required nasal continuous positive airway pressure (CPAP) during the first 48 hours, transitioned to nasal high-flow cannula on D6, and subsequently maintained spontaneous breathing in room air. They remained hemodynamically stable with normal urine output, renal function, electrolytes, and glycaemia. 

Due to increased serum bilirubin, both twins underwent phototherapy; twin A during DOL 3 to 5 and 7 to 8 (peak on D3) and twin B during DOL 4 to 6 (peak on D3), with normalization of bilirubin level. They received personalised parenteral nutrition until DOL 15, reached full enteral feeds on D16, and oral feeding autonomy by D29. 

Neurological evaluation showed, in twin A, bilateral grade I peri-intraventricular hemorrhage, and in twin B, the same findings plus a small left choroid plexus cyst. Both were discharged home on DOL 29 at postmenstrual age of 37 weeks+1 day, and scheduled follow-up in Developmental Paediatrics, Neonatology, Ophthalmology, Rheumatology, and Paediatric Cardiology.

At two months of age, in a Paediatric Rheumatology appointment, neither twin presented cutaneous lesions. At eight months, repeated autoimmune studies showed negative ANA, anti-dsDNA, and anti-U1-RNP antibodies, without any other analytic abnormalities (Tables 3, 4), and they were discharged. At five months, the Cardiology evaluation (electrocardiogram and echocardiogram) was normal; a repeat ECG was recommended at three years of age. At the present age (15 months), both infants show normal ophthalmologic screening (no retinopathy of prematurity), cranial ultrasound with complete resolution of hemorrhages, and adequate growth and psychomotor development.

**Figure 3:**
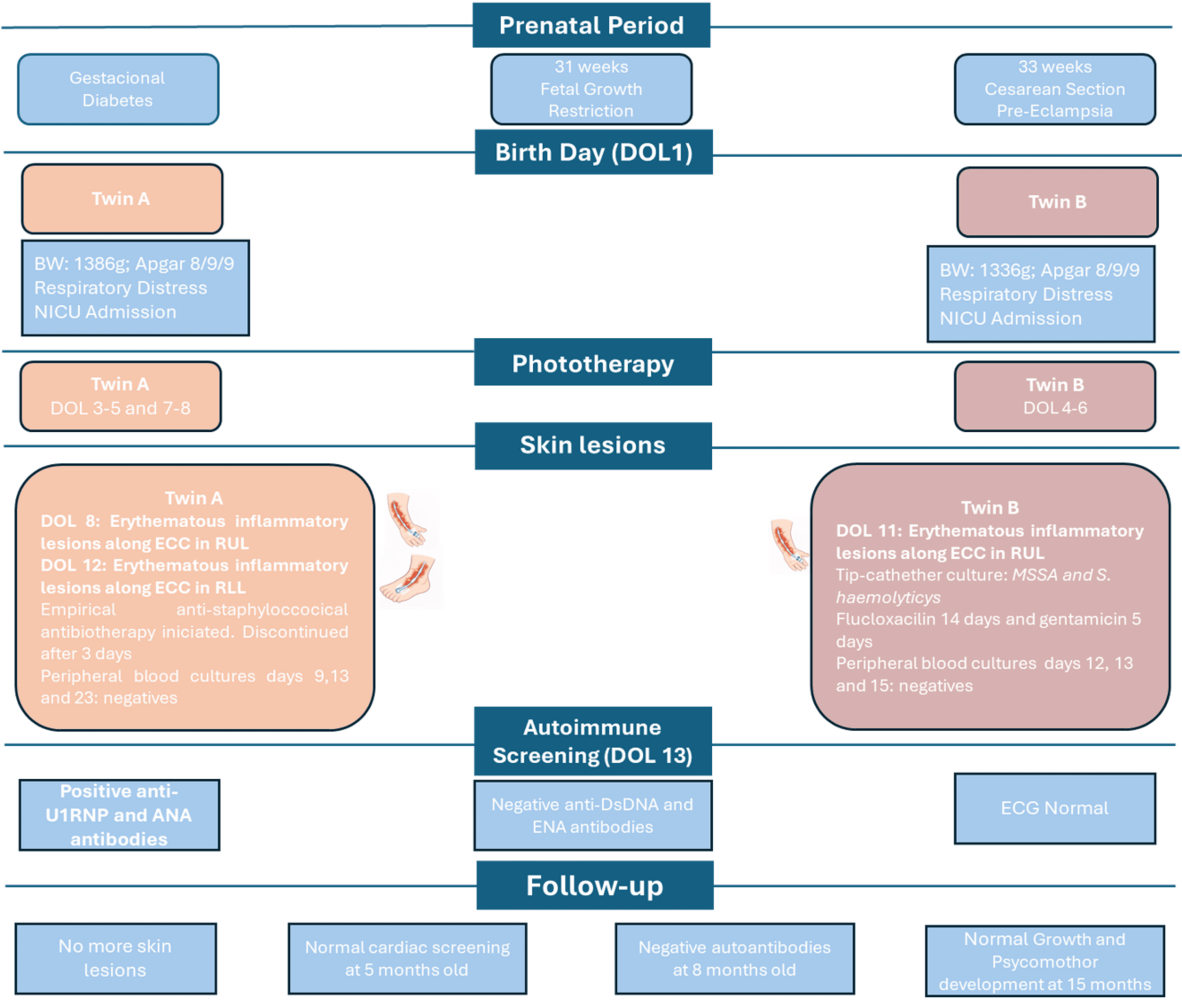
Timeline of clinical events in preterm twin girls with catheter-aligned cutaneous manifestations of neonatal lupus. ANA: antinuclear antibodies; anti-dsDNA: anti–double-stranded DNA antibodies; BW: birth weight; DOL: day of life; ECC: Epicutaneo-cava catheter; ECG: electrocardiogram; ENA: extractable nuclear antigen antibodies; MSSA: methicillin-susceptible *Staphylococcus aureus*; NICU: neonatal intensive care unit; RLL: right lower limb; RUL: right upper limb.

Figure 3 illustrates a timeline depicting the sequence of clinical events in the case presented.

Maternal investigation revealed the mother (30 years old, G0P0, blood group A, Rh-positive, previously healthy) had a family history of autoimmune disease (a maternal cousin with systemic lupus erythematosus (SLE) and a maternal aunt with systemic scleroderma). Following the neonatal findings, maternal testing demonstrated positive anti-U1RNP antibodies and ANA, though she did not pursue further evaluation.

## Discussion

NLE is a rare, passively acquired autoimmune syndrome. The primary autoantibodies involved are anti-Ro/SSA and/or anti-La/SSB, which are passively transferred across the placenta [2-4,8]. While these classical antibodies account for the vast majority of NLE cases, NLE is occasionally associated with anti-RNP antibodies and is regarded as a rare, distinct clinical subset that often presents solely with atypical cutaneous lesions [8-10,12]. Our case, involving BCBA twins with elevated anti-RNP antibodies is highly unusual, as published reports of NLE in twins are limited and, to our knowledge, with positive anti-RNP antibodies there are only two published reports: one with systemic severe disease in a mother with SLE with both anti-RNP and anti-dsDNA antibodies [7], and one with typical cutaneous manifestations at 45 days of life [12]; we believe that our paper is the first reported case with atypical erythematous lesions along the catheter trajectory. 

NLE results from the transplacental transfer of maternal IgG autoantibodies. The pathogenesis requires more than just the presence of these antibodies in the fetal circulation, suggesting roles for other antibodies, fetal genetic factors, and environmental stressors [1,2,4]. The key mechanism involves the translocation of typically intracellular antigens (like Ro/SSA and La/SSB) to the cell surface of various fetal cells, including cardiomyocytes and keratinocytes, during physiological apoptosis or subsequent to environmental stress. The binding of maternal autoantibodies to these surface antigens forms pathogenic immune complexes. This binding impairs the normal, non-inflammatory clearance of apoptotic cells, triggering an inflammatory cascade. The subsequent activation of macrophages leads to the secretion of proinflammatory cytokines, such as tumor necrosis factor (TNF), and profibrotic cytokines like transforming growth factor (TGF). This process ultimately results in tissue damage, fibrosis, and calcification, particularly in the cardiac conduction system [1,2,11]. While anti-Ro/SSA and anti-La/SSB are the primary agents in cardiac injury, anti-RNP antibodies target ribonucleoproteins involved in RNA processing. Their specific role is still largely undefined [8], although reactivity solely to U1RNP has been reported to be associated with cutaneous manifestations.

NLE manifestations are divided into irreversible (cardiac) and transient (non-cardiac) features [1-3,6,13], the latter resolving as maternal IgG antibodies clear from the infant's circulation, typically by six to eight months of age [1-4,6,9]. Regarding transient manifestations, the rash is the most frequent non-cardiac finding, seen in 4-16% of exposed infants [3]. It classically presents as erythematous annular lesions or arcuate macules, often found on sun-exposed areas like the periorbital region or the scalp. The lesions typically appear within the first few weeks after birth, usually around six weeks of age [12]. The skin lesions in NLE are self-limited and generally resolve without sequelae, with a reported mean duration of approximately 17 weeks and most cases resolving between six and eight months of age [11,13], although residual findings such as telangiectasia, dyspigmentation, or atrophy may persist in a minority of children [13]. Systemic manifestations include elevated liver enzymes, cholestasis, and transient hematologic cytopenias [1-5], such as thrombocytopenia and neutropenia, which were observed in twin A with subsequent recovery.

Previous reports of NLE associated with anti-U1RNP antibodies have characterized cutaneous manifestations as malar erythema and polycyclic plaques [14]. In contrast, the cutaneous presentation observed in our cases differed significantly in both morphology and distribution, manifesting as inflammatory lesions strictly confined to the trajectory of the epicutaneo-caval catheter. To our knowledge, there are no previous reports describing NLE skin lesions occurring along catheter trajectories in twins associated with anti-U1RNP antibodies. A similar pattern of lesions has been reported once in a preterm newborn with positive anti-SSA antibodies, supporting the plausibility of trauma-induced lesion localization in cutaneous neonatal lupus [15].

In this clinical context, catheter-associated infection represents an important differential diagnosis and cannot be entirely excluded, particularly given the isolation of microorganisms from catheter tips. Nonetheless, the strict confinement of the inflammatory changes to the catheter pathway, together with the absence of systemic signs of infection and the favorable clinical evolution, supports an alternative interpretation within the framework of a Koebner effect in cutaneous neonatal lupus.

The Koebner phenomenon describes the appearance of new skin lesions characteristic of an underlying dermatosis following trauma or nonspecific injury to previously unaffected skin. Although specific descriptions of Koebner-related lesions in neonatal lupus are scarce, trauma such as catheter insertion has been proposed as an environmental trigger capable of inducing local inflammatory responses, including the release of inflammatory mediators and heat shock proteins (Hsp70, TNF-α, IL-1, IL-6). In the context of NLE, such localized trauma may promote intracellular autoantigen exposure and amplify local inflammation, thereby facilitating the binding of circulating maternal autoantibodies and contributing to lesion development along the path of mechanical injury [16].

In addition, prior exposure to phototherapy for hyperbilirubinemia may have contributed, as light-based energy absorbed by keratinocytes can act as an environmental stress analogous to ultraviolet exposure, potentially inducing translocation of Ro/La/U1RNP antigens and triggering or exacerbating cutaneous NLE [9,12,13]. These two factors, mechanical trauma and phototherapy, may have served as environmental triggers superimposed upon the presence of circulating maternal autoantibodies.

The likelihood of an immune-mediated mechanism is strengthened by the synchronous appearance of identical lesions in both twins, both of whom had strongly positive anti-U1RNP antibodies, as well as by the improbability of infectious phlebitis arising in three separate catheters over a short period of time. The moderate transient neutropenia in one twin and the absence of systemic signs of infection further support a non-infectious etiology. Moreover, the spontaneous resolution of lesions and the disappearance of autoantibodies by eight months, consistent with the natural history of NLE, reinforce the diagnosis of an anti-U1RNP-mediated cutaneous process.

The most critical determinant of prognosis in NLE is the presence of cardiac abnormalities. Infants with no evidence of AV block at any stage, either in utero or at birth on ECG, are considered highly unlikely to develop cardiac disease later in life [2]. Although anti-RNP-only NLE is generally regarded as limited to cutaneous involvement and relatively cardioprotective [7,8], isolated reports describe transient first-degree block in the absence of anti-Ro/SSA and anti-La/SSB antibodies [17], as well as a case of advanced heart block associated solely with anti-RNP positivity [18]. In our case, an ECG was performed immediately when NLE was suspected, and both infants subsequently underwent cardiology assessment with ECG and echocardiography at two months of age, which were normal. Follow-up includes a repeat ECG scheduled at three years of age.

Children with NLE, even if only cutaneous, are at an increased risk of developing other autoimmune or rheumatic diseases later in life, such as juvenile idiopathic arthritis (JIA), psoriasis, or thyroid disease [1,8,13]. This is unclear for antiRNP solely positive antibodies [13]. Therefore, careful, long-term follow-up is recommended. 

Frequently, the mother is clinically asymptomatic when the neonate presents with characteristic manifestations, making the child’s diagnosis the first indication of maternal seropositivity [2,4,13]. The identification of maternal anti-RNP antibodies, particularly in the absence of anti-Ro and anti-La antibodies, holds specific diagnostic relevance, as anti-U1RNP is the defining antibody of mixed connective tissue disease and is also present in a subset of SLE and systemic sclerosis cases [3,17]. Accordingly, detection of anti-RNP antibodies in the neonate warrants maternal autoimmune testing and rheumatologic follow-up, given that approximately half of asymptomatic seropositive women eventually develop overt autoimmune symptoms [2]. In this case, the mother additionally had a family history of lupus and systemic sclerosis, and maternal testing revealed high-titter ANA and anti-RNP positivity. Although she declined further evaluation, the infants’ diagnosis provided the opportunity to identify previously unrecognized maternal autoimmunity.

As understanding of anti-U1RNP-associated NLE continues to evolve, documenting unusual manifestations such as those observed in these twins is essential to refining clinical suspicion and improving early recognition of this rare entity.

## Conclusions

This case describes a unique presentation of anti-U1RNP-associated neonatal lupus in preterm BCBA twins, manifesting as atypical inflammatory lesions strictly following the path of ECCs. This expands the recognized spectrum of anti-U1RNP-related cutaneous NLE and provides the first documentation in twins of a Koebner-like catheter-associated presentation. Importantly, the diagnosis in the newborns prompted the identification of previously unrecognized maternal autoimmunity, reinforcing the clinical relevance of neonatal findings for maternal health. Given the generally benign prognosis of anti-U1RNP NLE and the absence of cardiac involvement, long-term outcomes are expected to be favourable, although continued follow-up remains warranted due to the potential for later autoimmune manifestations.

## References

[1] Di Ludovico A, Rinaldi M, Mainieri F (2024). Molecular mechanisms of fetal and neonatal lupus: a narrative review of an autoimmune disease transferal across the placenta. Int J Mol Sci.

[2] Costa Cascais F, Fraga S, Sousa S, Pinto M (2021). Neonatal lupus: a clinical challenge. BMJ Case Rep.

[3] Gryka-Marton M, Szukiewicz D, Teliga-Czajkowska J, Olesinska M (2021). An overview of neonatal lupus with anti-Ro characteristics. Int J Mol Sci.

[4] Vanoni F, Lava SA, Fossali EF (2017). Neonatal systemic lupus erythematosus syndrome: a comprehensive review. Clin Rev Allergy Immunol.

[5] Silverman E, Jaeggi E (2010). Non-cardiac manifestations of neonatal lupus erythematosus. Scand J Immunol.

[6] Silver R, Craigo S, Porter F, Osmundson SS, Kuller JA, Norton ME (2023). Society for Maternal-Fetal Medicine consult series #64: systemic lupus erythematosus in pregnancy. Am J Obstet Gynecol.

[7] Lien J, Bhatti F (2025). Cardiopulmonary manifestations of neonatal lupus erythematosus: a case presentation in twins. Am J Med Sci.

[8] Heelan K, Watson R, Collins SM (2013). Neonatal lupus syndrome associated with ribonucleoprotein antibodies. Pediatr Dermatol.

[9] Sheth AP, Esterly NB, Ratoosh SL, Smith JP, Hebert AA, Silverman E (1995). U1RNP positive neonatal lupus erythematosus: association with anti-La antibodies?. Br J Dermatol.

[10] Su CT, Huang CB, Chung MY (2001). Neonatal lupus erythematosus in association with anti-RNP antibody: a case report. Am J Perinatol.

[11] Buyon JP (2025). Neonatal lupus: epidemiology, pathogenesis, clinical manifestations, and diagnosis. UpToDate.

[12] Matucci-Cerinic C, Viglizzo G, Ravelli A, Occella C (2021). Neonatal lupus erythematosus in dizygotic twins with anti-RNP antibodies. Clin Exp Rheumatol.

[13] Neiman AR, Lee LA, Weston WL, Buyon JP (2000). Cutaneous manifestations of neonatal lupus without heart block: characteristics of mothers and children enrolled in a national registry. J Pediatr.

[14] Dugan EM, Tunnessen WW, Honig PJ, Watson RM (1992). U1RNP antibody-positive neonatal lupus. A report of two cases with immunogenetic studies. Arch Dermatol.

[15] Franco C, Fortunato F, Maria AT, Marçal M (2018). Recurrent central catheter complications in a newborn: can we blame neonatal lupus?. BMJ Case Rep.

[16] Ueki H (2005). Koebner phenomenon in lupus erythematosus with special consideration of clinical findings. Autoimmun Rev.

[17] Acherman RJ, Friedman DM, Buyon JP, Schwartz J, Castillo WJ, Rollins RC, Evans WN (2010). Doppler fetal mechanical PR interval prolongation with positive maternal anti-RNP but negative SSA/Ro and SSB/La auto-antibodies. Prenat Diagn.

[18] Izmirly PM, Halushka MK, Rosenberg AZ (2017). Clinical and pathologic implications of extending the spectrum of maternal autoantibodies reactive with ribonucleoproteins associated with cutaneous and now cardiac neonatal lupus from SSA/Ro and SSB/La to U1RNP. Autoimmun Rev.

